# Supplementary material for: Effects of self-selected versus motivational music on lower limb muscle strength and affective state in middle-aged adults
Source: PeerJ. 2022 Jul 22;10:e13795. doi: 10.7717/peerj.13795 (PMC9310772; doi:10.7717/peerj.13795)
Supplement: Supplemental Information 2 — BPM: beats per minute [file peerj-10-13795-s002.pdf]

SONG TITLE	ARTIST	BPM
30/30-150	Stone Sour	163
50 Special	The pop moon group	80
A mano a mano	Rino Gaetano	88
A Sky Full of Stars	Coldplay	125
Acqua azzurra, acqua chiara	Lucio Battisti	92
Aeroplane	Red Hot Chili Peppers	101
Aida	Rino Gaetano	73
All Along the Watchtower	Jimi Hendrix	113
Always	Bon Jovi	71
Andromeda	Elodie	122
Another Day in Paradise	Phil Collins	102
Back in Black	AC/DC	94
Back in Black	AC/DC	94
Berta Filava	Rino Gaetano	114
Better think again	Submersed	100
Billie Jean	Michael Jackson	117
Bocca di rosa	Fabrizio De André	118
Bohemian Rhapsody	Queen	72
Born in the U.S.A.	Bruce Springsteen	122
Born tu Run	Bruce Springsteen	73
Breaking Me	Topic & A7S	122
Bring me to life	Evanescence	95
Cake by the Ocean	DNCE	119
Can’t Stop	Red Hot Chili Peppers	91
Can't get you out of my head	Kylie Minogue	63
Children	Robert Miles	137
Coming for You	The Offspring	139
Cosa ti aspetti da me	Loredana Bertè	93
Counting Stars	OneRepublic	122
Crazy	Aerosmith	81

Dance with the dead	Axxis	180
Dancing in the dark	Bruce Springsteen	149
Dangerous	David Guetta feat. Sam Martin	92
Diamonds	Sam Smith	104
Dolcenera	Fabrizio De André	126
Don Raffaè	Fabrizio De André	128
Don’t Stop Me Now	Queen	156
Don’t worry	Madcon feat. Ray Dalton 71	123
Don't stop me now	Queen	156
Don't you want me	The Human League	118
Duality	Slipknot	144
E cantava le canzoni	Rino Gaetano	116
El Pueblo Unido jamas sera vencido	Banda Bassotti	140
Eye of the tiger	Survivor	109
Eye of the Tiger	Survivor	109
Figli del Vento	Raphael Gualazzi	90
Fire Your Guns	AC/DC	99
Fly Away	Tones And I	125
Fortunate Son	Creedence Clearwater Reviva	133
Fuck authority	Pennywise	102
Galway Girl	Ed Sheeran	100
Gente di mare	Umberto Tozzi	87
Gianna	Rino Gaetano	106
Glory Days	Bruce Springsteen	117
Got The Time	Anthrax	128
Happy song	Bring me the horizon	86
Head & Heart	Joel Corry feat. MNEK	123
Hey Ya	Out Kast	80
Highway star	Deep purple	174
Highway to Hell	AC/DC	116
I just can't get enough	Modern Rock Player	127

I was made for loving'you	Kiss	128
I Wish	Stevie Wonder	106
Il mio nome è nessuno	Ennio Morricone	112
Il posto dei santi	Negramaro	60
I'm a believer	Smash Mouth	82
Immigrant song	Led Zeppelin	113
In the end	Linkin Park	105
I've got the fire	Kissin' Dynamite	117
Killing in the name on	Rage against the machine	89
La canzone del sole	Lucio Battisti	93
La mia banda suona il rock	Ivano Fossati	106
Last Train Home	Pat Metheny Group	80
Let there be rock	AC/DC	182
Like a Rolling Stones (Live)	The rolling stones	103
Living in America	James Brown	114
Locked out of heaven	Bruno Mars	72
Locomotive Breath	Jethro Tull	125
L'ombelico del mondo	Jovanotti	110
Lose Control	Meduza, Becky Hill, Goodboys	124
Losing my religion	R.E.M.	63
Losing my Religion	R.E.M.	63
Love is Gone	David Guetta feat Chris Willis	128
Love the way you lie	Eminem feat. Rihanna	87
Lovefool	twocolors	123
Lucky man	The Verve	76
Margarita	Elodie feat. Marracash	96
Metalingus	Alter Bridge	110
More than a feeling	Boston	109
My Immortal	Evanescence	79
My plague	Slipknot	89
Nel letto di Lucia	Rino Gaetano	103

New Year’s Day	U2	133
No Easy Way Out	Rocky Soundtrack	126
No Roots	Alice Merton	116
One step beyond	Madness	155
One Step Beyond	Madness	155
Paint it black	The rolling stones	159
Papaoutai	Stromae	116
Physical	Dua Lipa	74
Preachin' Blues	Larkin Poe	86
Push the button	Sugababes	63
Renegades	X Ambassadors	90
Rise today	Alter Bridge	159
Shock to the system	Billy Idol	85
Should I stay or Should I go	The Clash	113
Smoke on the water	Deep purple	114
Sonne	Rammstein	75
Stairway to Heaven	Led Zeppelin	82
Starway to heaven	Studio Sound Group	81
Sugar	Maroon 5	60
Sweet child O'Mine	Guns N'Roses	125
Tell me if you wanna go home	Keira Knightley	72
The final countdown	Europe	118
The kids aren't alright	The Offspring	100
Think	Aretha Franklin	55
Till I collapse	Eminem	86
Too old to rock'n' Roll: too young to die!	Jethro Tull	75
Umbrella	Rihanna	87
Walk this way	RUN-DMC	106
We are the champions	Queen	95
We will rock you	Queen	81
Welcome to the Jungle	Guns N'Roses	124

We're Gonna Win	Bryan Adams	172
Who made who	AC/DC	125
Whole Lotta Love	Led Zeppelin	90
Zitti e buoni	Maneskin	103
